# The Landscape of Biomarkers in ICU‐Associated AKI: From Protein Markers to Cell‐Free Nucleic Acids

**DOI:** 10.1002/jcla.70311

**Published:** 2026-08-05

**Authors:** Qi Zhu, Feng Yang, Hua Zheng, Xinxin Guo, Zhijin Chen, Limin Chen, Daoli Wang, Jiaqi Wang, Xinmeng Wang, Zhiyuan Huang, Zhenhua Ji

**Affiliations:** ^1^ Department of Emergency Central Hospital Affiliated to Shenyang Medical College Shenyang China; ^2^ Department of Radiology Central Hospital Affiliated to Shenyang Medical College Shenyang China; ^3^ Department of Nephrology Central Hospital Affiliated to Shenyang Medical College Shenyang China

**Keywords:** AKI, biomarkers, cfDNA, ICU, mtDNA, sepsis

## Abstract

**Background:**

Acute kidney injury (AKI) affects approximately 30%–60% of intensive care unit (ICU) admissions, but early detection remains difficult because creatinine‐ and urine‐output‐based KDIGO criteria are delayed and confounded in critical illness.

**Objective:**

To summarize the biomarker landscape for ICU‐associated AKI, focusing on kidney‐targeted injury/stress proteins and circulating cell‐free DNA (cfDNA), including mitochondrial DNA (mtDNA).

**Methods:**

PubMed/MEDLINE and Embase were searched from inception to December 31, 2025, using terms related to AKI, critical illness, NGAL, KIM‐1, IL‐18, L‐FABP, [TIMP‐2]·[IGFBP7], cfDNA, mtDNA, and damage‐associated molecular patterns. Adult ICU studies reporting diagnostic performance, risk stratification, or clinically relevant outcomes were prioritized, especially in sepsis, cardiac surgery, shock, and trauma.

**Results:**

NGAL, KIM‐1, IL‐18, L‐FABP, and [TIMP‐2]·[IGFBP7] often rise before creatinine or urine‐output changes, providing early kidney‐proximal signals of tubular injury or stress. cfDNA/mtDNA instead reflects systemic cell death, mitochondrial damage, and innate immune activation, capturing multi‐organ injury and adding prognostic information beyond clinical scores and creatinine. Across ICU phenotypes, combining clinical risk, an early kidney‐targeted marker, and cfDNA/mtDNA may improve identification of patients at risk for severe AKI, renal replacement therapy, and death.

**Conclusions:**

Multimarker strategies may widen the diagnostic window and sharpen prognostication in ICU‐associated AKI. Routine implementation will require standardized pre‐analytical handling, assay harmonization, biomarker‐guided pragmatic trials, and trajectory‐based decision tools that demonstrate clinical benefit.

## Graphical abstract

Acute kidney injury (AKI) in critically ill patients—particularly in sepsis—often develops early and is missed by creatinine/urine output–based criteria, delaying risk stratification and timely intervention. This review synthesizes current evidence on established and emerging AKI biomarkers (e.g., NGAL, KIM‐1, [TIMP‐2]·[IGFBP7]) alongside circulating cell‐free DNA/mitochondrial DNA, highlighting their diagnostic and prognostic value, practical limitations, and how multimarker strategies may improve ICU decision‐making.

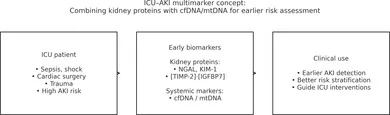

## 1 Introduction

Acute kidney injury (AKI) in the intensive care unit (ICU) is a common and life‐threatening complication, affecting roughly 30%–60% of critically ill patients [1]. ICU‐AKI is associated with high mortality and morbidity. In severe cases (e.g., Stage 3 AKI requiring dialysis), in‐hospital mortality rates can exceed 50% [2, 3]. Many patients with ICU‐AKI require renal replacement therapy (RRT) during their ICU stay, and those who survive remain at risk for long‐term sequelae such as chronic kidney disease (CKD) and cardiovascular events [4, 5]. To quantify the burden of AKI, composite outcomes like major adverse kidney events at 30 days (MAKE30) are often used: MAKE30 encompasses the incidence of death, new RRT initiation, or persistent renal dysfunction within 30 days of AKI [6].

Currently, AKI is defined by the Kidney Disease Improving Global Outcomes (KDIGO) criteria based on increases in serum creatinine (sCr) or oliguria over time, which serve as surrogate markers of glomerular filtration [7]. However, these traditional indices have important limitations in the ICU setting. Serum creatinine is an insensitive and delayed indicator of injury—its rise may lag 24–72 h behind an acute insult due to slow kinetics and renal reserve, and it can be confounded by fluid balance, liver dysfunction, or low muscle mass [8]. A post hoc analysis of ICU patients showed that adjusting sCr for positive fluid balance revealed “hidden” AKI in nearly 18% of cases that would have been missed by unadjusted creatinine levels [9]. Conversely, sCr can overestimate kidney injury in certain ICU scenarios (e.g., patients on cimetidine or trimethoprim therapy) by reducing creatinine secretion [8, 10]. The urine output criterion is also problematic. Urine volume often poorly correlates with true GFR and may remain normal until profound dysfunction is present. Oliguria can occur as an appropriate physiological response (e.g., to hypovolemia or stress) and, on the other hand, diuretics can increase urine output in AKI without improving renal function [11]. Thus, sole reliance on sCr and urine output tends to detect AKI only at a late stage in many ICU settings and can overlook “subclinical” injury. This diagnostic delay is thought to impede timely intervention and partly explain the persistently poor outcomes of ICU‐AKI [12].

Recognizing these issues, recent research has focused on identifying biomarkers that signal kidney injury or stress earlier and more specifically, thereby improving diagnostic timing and risk stratification. The discovery of such biomarkers—spanning from proteins released by injured tubular cells to circulating fragments of DNA from dying cells—has opened the door to detecting AKI in its incipient stages (even before KDIGO criteria are met) and to refining prognostication [13]. In this mini‐review, we outline the evolving landscape of AKI biomarkers in the ICU, highlighting how the field has progressed from traditional protein‐based indicators like NGAL to emerging nucleic acid markers such as cell‐free DNA (cfDNA) and mitochondrial DNA (mtDNA). The goal is to illustrate how these advances may enable earlier diagnosis and more accurate prediction of outcomes in ICU‐associated AKI.

To inform this focused narrative review, we searched PubMed/MEDLINE and Embase (from inception to December 31, 2025) using combinations of terms related to acute kidney injury, intensive care/critical illness, and biomarker concepts (e.g., NGAL, KIM‐1, IL‐18, L‐FABP, [TIMP‐2]·[IGFBP7], cell‐free DNA, cfDNA, mitochondrial DNA, mtDNA, and damage‐associated molecular patterns (DAMPs)). We prioritized studies in adult ICU populations (sepsis, shock, cardiac surgery, and trauma) reporting diagnostic performance, risk stratification, or clinically relevant outcomes, and we preferentially cited recent meta‐analyses/systematic reviews and multicenter cohorts when available. Reference lists of key articles were manually screened to identify additional eligible studies.

## 2 Biomarker Landscape and Clinical Window in ICU‐AKI

### 2.1 Classification Framework for AKI Biomarkers

**FIGURE 1:**
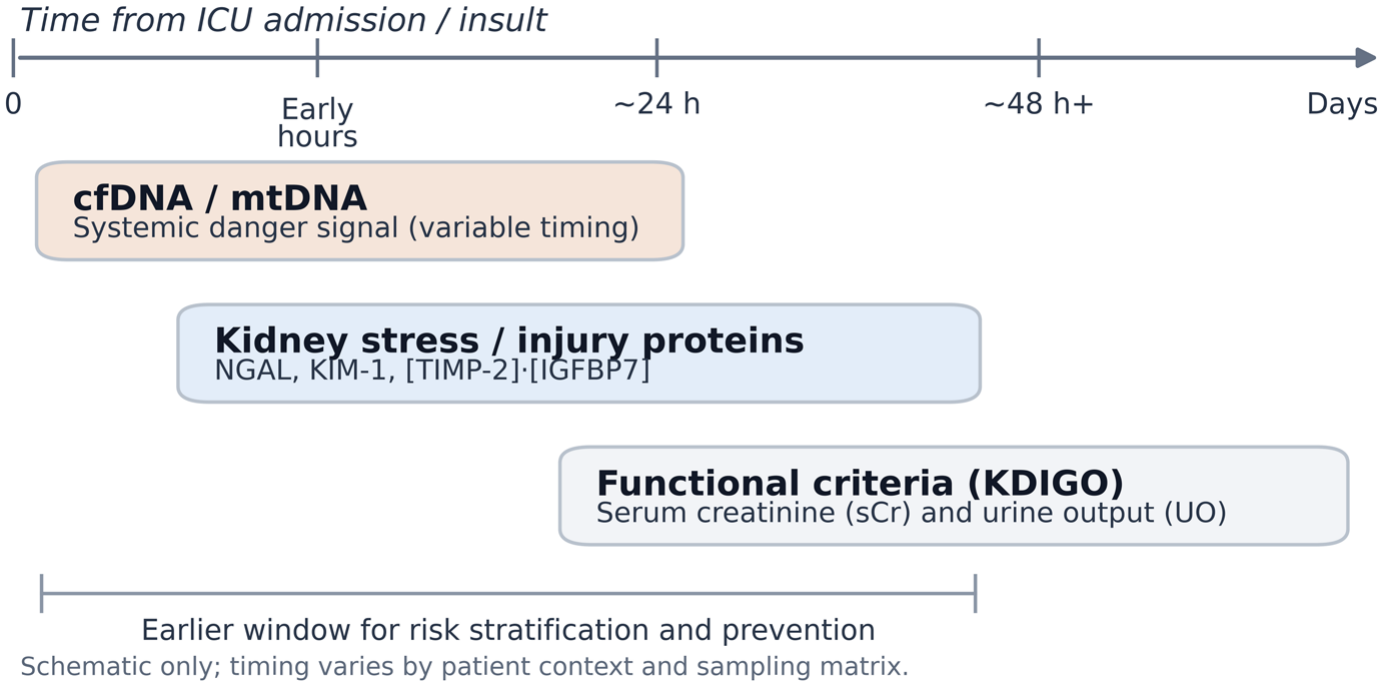
Biomarker clinical window in ICU‐AKI.

The heterogeneous nature of ICU‐AKI has led to a conceptual framework that classifies biomarkers by the aspect of injury or function they reflect [8]. Functional biomarkers primarily indicate changes in glomerular filtration rate—e.g., serum creatinine itself, cystatin C, or novel filtration markers like proenkephalin reflect real‐time excretory function. These rise when GFR declines, but because of renal reserve and kinetic delays, functional markers may not increase until well after an injury has occurred [14, 15, 16]. Injury biomarkers, in contrast, signify structural damage to kidney cells (often tubular epithelium). Classic examples include neutrophil gelatinase‐associated lipocalin (NGAL) [17], kidney injury molecule‐1 (KIM‐1) [18], liver‐type fatty acid–binding protein (L‐FABP) [19], and interleukin‐18 (IL‐18) [20], which are released or upregulated in response to tubular cell injury or inflammation. Stress biomarkers denote early cellular distress that precedes overt cell death. Notably, the cell cycle arrest proteins tissue inhibitor of metalloproteinases‐2 (TIMP‐2) and insulin‐like growth factor binding protein‐7 (IGFBP7) fall into this category—they are released from stressed tubule cells to signal G1 cell‐cycle arrest, essentially serving as an alarm of impending injury [21]. Finally, “danger” signals or damage‐associated molecular patterns (DAMPs) are molecules released during cell injury or necrosis that trigger immune responses. This group includes extracellular DNA fragments, mitochondrial DNA, histones, HMGB1, and other intracellular constituents that appear in the circulation during severe tissue injury [22]. While not kidney‐specific, these DAMPs provide a systemic readout of injury and have been implicated in the pathogenesis of sepsis‐related AKI and multi‐organ dysfunction. By classifying biomarkers into functional, injury, stress, and danger signal categories, one can map them onto the temporal course of AKI—from early stress (with risk of injury) through overt cellular damage and loss of function—thereby defining a “clinical window” for potential intervention at each stage (Figure 1).

### 2.2 Classical and Emerging Biomarkers for ICU‐AKI

Over the past decade, numerous biomarkers spanning these categories have been studied for their utility in early diagnosis and prognostication of ICU‐AKI (Table 1). Among the classical injury biomarkers, NGAL has been one of the most extensively investigated. NGAL is released from neutrophils and upregulated in injured tubular cells; it can be detected in blood or urine within a few hours of kidney injury. Clinical studies have shown that an isolated rise in urine NGAL can presage AKI development days before a creatinine rise—for instance, children with elevated NGAL had a significantly higher risk of developing AKI despite normal sCr in the first 48 h [24]. Meta‐analyses encompassing thousands of patients in diverse settings confirm that NGAL is a useful early indicator of AKI [25, 26]. KIM‐1 is another prototypical injury marker: A transmembrane glycoprotein highly expressed in dedifferentiated proximal tubule cells after ischemic or toxic injury [27]. Urinary KIM‐1 levels correlate with the extent of tubular damage and have shown predictive value for AKI, especially in ischemic contexts such as cardiac surgery [28]. IL‐18, a pro‐inflammatory cytokine produced by macrophages and proximal tubule cells, is released into the urine during acute tubular injury; it was originally identified as a marker for ischemic AKI (e.g., in post‐operative settings) and has since been associated with sepsis‐related AKI as well [29]. Liver‐type FABP is a cytosolic protein abundant in proximal tubule cells that is released when those cells undergo ischemia or oxidative stress; urinary L‐FABP has demonstrated early diagnostic capability in settings like sepsis and cardiac surgery, and its levels tend to reflect the severity of renal ischemic stress [30]. These classical biomarkers of injury/inflammation have not only shown promise in detecting AKI earlier than the rise in creatinine, but in some studies, their magnitude also stratified risk. For example, concurrent measurement of a panel of NGAL, IL‐18, KIM‐1, and L‐FABP has been explored to predict the likelihood of adverse outcomes or persistent AKI after cardiac surgery [31].

**TABLE 1:** Representative primary studies on AKI biomarkers relevant to ICU practice.

Biomarker(s)	Study design & population	N	Sampling window	Primary/Key endpoints	Main quantitative findings	AKI/KRT/Mortality signal	References
**[TIMP‐2]·[IGFBP7]**	Randomized, open‐label ED triage vs. standard care in adults at risk of AKI	100 (randomized)	At ED presentation; repeat up to 24–48 h	Primary: Moderate–severe AKI ≤ 24 h; Secondary: ΔSCr to 48 h, KRT, ICU/LOS	No difference in primary endpoint; lower day‐2 SCr in intervention; KRT 0/50 vs. 3/50 (*p* = 0.09)	Neutral on AKI; trend to fewer KRT events; no clear mortality effect	[14]
**Urine NGAL**	Prospective ED cohort; adults evaluated for AKI	635	Single ED sample	AKI diagnosis; composite (dialysis/ICU admission/mortality)	NGAL distinguished AKI from other causes; at 130 μg/g Cr, NGAL strongly associated with composite outcome (OR 24.7, 95% CI 7.69–79.4)	Predicts need for intensive care and dialysis/mortality composite	[15]
**Urine IL‐18; plasma & urine NGAL**	Prospective cardiac‐surgery cohort (TRIBE‐AKI, adults)	1,219	First 6 h post‐op (serial)	AKI (doubling SCr or dialysis); LOS, ICU stay, dialysis, death	Highest‐quintile IL‐18: OR 6.8 for AKI; plasma NGAL: OR 5.0; adding IL‐18 or plasma NGAL to the clinical model improved AUC to 0.76 and 0.75	Higher odds of dialysis or death and longer LOS/ICU	[16]
**Urine KIM‐1 & L‐FABP**	Multicenter cardiac‐surgery cohorts (adults & children)	1,219 adults; 311 children	L‐FABP peaks ≤ 6 h; KIM‐1 peaks ~post‐op day 2	Post‐op AKI; incremental value with panels	First post‐op L‐FABP not independently associated with AKI after adjustment; both markers lost significance when NGAL/IL‐18 included; best multi‐marker panel AUC≈0.78	Limited incremental clinical utility alone; value mainly in panels	[17]
**Serum cystatin C**	Prospective ICU high‐risk cohort	85 (44 ARF, 41 controls)	Daily sampling	Early ARF detection (RIFLE R/I/F); prediction of RRT	Detected ARF **1.5 ± 0.6 days earlier** than creatinine; AUC for R‐criteria detection: 0.82 (day −2) and 0.99 (day 0); predicted later RRT (AUC 0.69–0.76 depending on day)	Earlier detection; moderate prediction of subsequent RRT	[18]
**Plasma proenkephalin A 119–159 (penKid)**	Prospective ED sepsis cohort	588	At ED admission	AKI ≤ 48 h and ≤ 7 d; renal SOFA progression; MOF; 28‐day mortality	Predicted AKI, but associations **attenuated after eGFR** adjustment; remained predictive of renal SOFA progression, MOF, and 28‐day mortality	Independent mortality and organ‐failure signal; AKI prediction weaker after eGFR	[19]
**Plasma suPAR**	Three prospective cohorts (observational + trial cohort)	3,827	At hospital/ICU admission	7‐day AKI; AKI or death	Highest‐tertile suPAR independently associated with AKI (adj. OR 2.66) and AKI or death (adj. OR 2.29); dose–response across cohorts	Strong, independent risk signal for AKI and AKI/death	[20]
**Urine KIM‐1, NGAL, N‐acetyl‐β‐D‐glucosaminidase (NAG)**	Prospective cardiac‐surgery cohort	90	Immediate post‐op & 3 h	Early AKI (AKIN) discrimination	AUC (immediate/3 h): KIM‐1 0.68/0.65; NAG 0.61/0.63; NGAL 0.59/0.65; **combined biomarker panel** AUC 0.75/0.78; **early post‐op subgroup** AUC 0.80/0.84	Panels outperform single markers; early post‐op discrimination is better	[21]
**Urinary mitochondrial DNA (UmtDNA)**	Human cardiac‐surgery cohort (SAKInet) + mouse I/R model	111	~1 day post‐op (median)	AKI progression (stable→progressive); mechanistic link to mitochondrial injury	Higher UmtDNA in **progressive** vs. stable/no AKI; ROC AUC 0.72 for progression; independent of creatinine and urinary angiotensinogen; in mice, UmtDNA tracked ischemia time and predicted AKI (AUC 0.88)	Human: Prognostic for **worsening AKI**; not a mortality/KRT study	[22]
**Plasma cell‐free DNA (cfDNA)**	Prospective on‐pump cardiac‐surgery cohort (single‐center)	58	Pre‐op; post‐op days 1–5 (serial)	Post‐op (late) AKI	**Day‐3 cfDNA AUC≈0.80** for late AKI; cfDNA trajectory outperformed several early markers for late events	Prognostic for late AKI; not a mortality/KRT study	[23]

In addition to direct injury markers, cellular stress markers have entered clinical use. The combination urine test for [TIMP‐2]·[IGFBP7] (commercialized as NephroCheck) exemplifies this category [32]. TIMP‐2 and IGFBP7 are proteins secreted by stressed or injured tubular cells to induce cell‐cycle arrest, thereby preventing cell division during sublethal injury. An elevated urinary [TIMP‐2]·[IGFBP7] indicates that the kidneys are under significant stress before irreversible damage occurs [33]. In critically ill adults, this test has been shown to predict the development of moderate‐to‐severe AKI (KDIGO stage 2–3) within 12–24 h of sample collection [34]. For instance, two large meta‐analyses reported an AUC of ~0.80 for [TIMP‐2]·[IGFBP7] in forecasting imminent AKI in high‐risk cohorts [35, 36]. It is also one of the first biomarker tools to receive regulatory approval, as the FDA and EMA approved it for AKI risk assessment in ICU patients, given its ability to stratify patients by risk [37, 38]. Clinically, [TIMP‐2]·[IGFBP7] has been used to identify high‐risk ICU patients for enrollment in trials of early intervention strategies [39]. While this has enhanced our ability to anticipate AKI, it is important to note that whether biomarker‐guided care improves patient outcomes remains an active area of investigation. Barriers such as cost, limited availability, and the need for effective early treatments have so far tempered the impact of these tests in routine practice.

To provide quantitative context for commonly used injury and stress biomarkers, Table 2 summarizes representative systematic reviews and meta‐analyses reporting pooled diagnostic performance metrics.

**TABLE 2:** Representative meta‐analyses of key biomarkers relevant to ICU‐associated AKI.

Biomarker (specimen)	Population/setting	Studies/patients	Pooled performance (examples)	Reference (add via EndNote)
NGAL (urine/plasma)	Mixed at‐risk cohorts incl. ICU; clinical lab platforms	Systematic review & meta‐analysis (individual‐study data)	AUC ~0.75–0.86 depending on specimen and outcome (severe AKI vs. AKI requiring dialysis)	[40]
[TIMP‐2]·[IGFBP7] (urine)	Adults; mixed settings include critical illness	Meta‐analysis (to March 2017)	AKI: SEN 0.83, SPE 0.72, AUC 0.86; Stage ≥ 2 AKI: SEN 0.92, SPE 0.63, AUC 0.88	[41]
KIM‐1 (urine)	Hospital‐based at‐risk cohorts (incl. cardiac surgery and ICU)	11 studies/2,979 patients	SEN 0.74, SPE 0.86, AUC 0.86	[42]
IL‐18 (urine)	Various settings incl. ICU, ED, and cardiac surgery	11 studies/2,796 patients	SEN 0.51, SPE 0.79, AUC 0.77	[43]
L‐FABP (urine/plasma)	Variable settings incl. ICU, surgery, and contrast exposure	27 studies	SEN 0.74, SPE 0.78, AUC 0.82	[44]
Cystatin C (serum/urine)	Mixed settings; early measurement after insult or ICU admission	Meta‐analysis (13 studies)/3,336 patients	Serum: SEN 0.86, SPE 0.82, AUROC 0.87; Urine: AUROC 0.67	[45]

Abbreviations: AKI‐D, AKI requiring dialysis. Performance estimates and settings vary across studies; see the cited meta‐analyses for details; AUC/AUROC, area under the (receiver operating characteristic) curve; SEN, sensitivity; SPE, specificity.

## 3 From Proteins to Nucleic Acids

### 3.1 Biological Origin and Pathophysiological Relevance of cfDNA/mtDNA

Cell‐free DNA (cfDNA) in critical illness originates from multiple cellular processes. Cells undergoing necrosis or pyroptosis release large DNA fragments due to loss of membrane integrity, whereas apoptosis yields smaller nucleosomal DNA fragments typically enclosed in apoptotic bodies [46]. In infection and inflammation, neutrophils undergo NETosis, an active form of cell death where neutrophil extracellular traps (NETs) composed of chromatin are expelled to trap pathogens. These processes all contribute to elevated cfDNA in the circulation during critical illness [47]. Notably, NET release, while part of host defense, can induce a “thrombo‐inflammatory” state, promoting microvascular thrombosis and organ injury [48]. Beyond passive release in cell death, certain cells may actively secrete DNA via exosomes or microparticles; e.g., activated platelets can release mitochondrial DNA (mtDNA) into the bloodstream, potentially amplifying inflammation in trauma and sepsis [49].

Importantly, mitochondrial DNA—a circular, bacterial‐origin DNA—has unique immunological significance. Mitochondrial DNA contains unmethylated CpG motifs resembling bacterial DNA, which are recognized by pattern recognition receptors [50]. When cellular stress or injury damages mitochondria, mtDNA can leak into the cytosol or extracellular space. Free cytosolic mtDNA acts as a potent danger‐associated molecular pattern (DAMP), triggering innate immune sensors [51]. It is detected by endosomal Toll‐like receptor 9 (TLR9), which binds CpG‐rich DNA and activates inflammatory NF‐κB signaling, and by cytosolic DNA‐sensing pathways such as the cGAS–STING axis and the NLRP3 inflammasome [50, 52, 53]. Through these pathways, mtDNA evokes production of cytokines (e.g., IL‐6, TNFα) and mediators like IL‐1β that drive inflammation. Notably, Tsuji et al. [54] demonstrated in a murine sepsis model that extracellular mtDNA engaging TLR9 contributes to cytokine release and kidney injury. Likewise, oxidized mtDNA can activate the NLRP3 inflammasome in renal cells, linking mitochondrial damage to caspase‐1 activation and IL‐1β release. In contrast to apoptosis (which is immunologically quiet and releases minimal DAMPs), unregulated cell death leads to abundant cfDNA/mtDNA that can perpetuate inflammation [46]. These DAMP effects are highly relevant in critical illness: Circulating mtDNA levels often remain elevated long after the initial insult, suggesting a role in sustaining systemic inflammatory response syndrome (SIRS) [55, 56]. In sepsis and acute kidney injury (AKI), mtDNA and other DAMPs drive a self‐amplifying cycle of inflammation, endothelial dysfunction, and organ damage. For instance, mitochondrial DAMPs have been implicated in sepsis‐associated microvascular dysfunction and tubular injury, as blocking TLR9 pathways has attenuated kidney damage in preclinical models [57]. In summary, cfDNA (including nuclear and mitochondrial components) serves not just as a marker of cell injury but as an active participant in pathophysiology: It links widespread cell death to immune activation. This is particularly pertinent in sepsis‐related AKI, where high DAMP levels (like mtDNA) correlate with the severity of inflammation and kidney dysfunction. Clinical and experimental data show that trauma or infection can cause surges in plasma mtDNA that correlate with the severity of organ failure. Thus, mechanistically, the shift “from proteins to nucleic acids” represents a paradigm where cfDNA/mtDNA are both biomarkers of cellular injury and mediators that can exacerbate systemic inflammation and organ injury in the ICU setting.

### 3.2 Clinical Evidence of cfDNA/mtDNA in ICU‐AKI

An increasing number of clinical studies over the past decade have evaluated cfDNA and mtDNA as biomarkers for ICU‐associated AKI. Several investigations suggest that cfDNA levels rise early in critical illness and may predict AKI development before or alongside traditional measures. In septic ICU patients, those who went on to develop AKI showed significantly higher plasma cfDNA at admission than those who did not [58]. Clementi et al. [59] observed that among septic patients, those who subsequently developed AKI (especially severe AKI requiring renal replacement therapy) had elevated cfDNA compared to those without AKI. Similarly, in a cohort of cardiac surgery patients—a population at high risk for AKI—serial cfDNA measurements improved early detection of kidney injury. In one study of 58 cardiac surgery patients, cfDNA levels on postoperative day 1–3 were significantly higher in patients who eventually developed AKI, even when serum creatinine was not yet markedly changed [23]. Notably, by postoperative day 3, cfDNA emerged as the strongest predictor of AKI (AUC≈0.80), outperforming classic biomarkers like neutrophil gelatinase‐associated lipocalin (NGAL, AUC≈0.70) and even creatinine [23]. This suggests that cfDNA can reflect ongoing renal injury earlier or more sensitively in certain contexts. However, timing is crucial, as in the same study, NGAL was superior for identifying very early (within 24 h) AKI, whereas cfDNA excelled for AKI manifesting a bit later (2–3 days post‐insult) [23]. Another small study in critically ill trauma and post‐surgery patients found that plasma cfDNA on ICU day 1 was significantly higher in those who developed sepsis or complications than in uncomplicated cases [60]. The ability of cfDNA to indicate extensive tissue injury may explain these findings—patients with higher initial cfDNA likely have greater injury burden, portending subsequent organ dysfunction, including AKI. In addition to plasma, urinary mtDNA has shown promise in early AKI detection. Urine mtDNA levels rise in patients with AKI and correlate with conventional biomarkers (creatinine, NGAL, KIM‐1) and inflammatory cytokines, reflecting local renal‐cell mitochondrial injury [51]. Such evidence positions cfDNA/mtDNA as potential early‐warning indicators, complementary to protein markers like NGAL or the cell‐cycle arrest markers (TIMP‐2·IGFBP7) currently used for AKI risk stratification.

Beyond diagnosis, the prognostic value of cfDNA/mtDNA in ICU‐AKI is supported by multiple clinical studies. High circulating cfDNA consistently tracks with worse outcomes in the critically ill, including need for renal replacement therapy (RRT) and mortality (e.g., higher admission cfDNA in ICU non‐survivors and in septic patients who went on to require CRRT) [59]. In sepsis‐associated AKI, a cohort from Zhongshan Hospital reported that patients with serum cfDNA ≥ 855 ng/mL had markedly lower Three‐month survival than those below this threshold; in multivariable models, elevated cfDNA carried a > 2‐fold hazard of death [61]. A recent prospective study further showed that septic patients with AKI had higher nuclear and mitochondrial cfDNA on day 3 of sepsis; cfDNA levels were higher in those who progressed to RRT, and adding cfDNA to creatinine‐based or APACHE II models improved prediction (AUC ~0.88 for RRT and~0.86 for 28‐day mortality) [46]. After cardiac surgery, persistently elevated cfDNA tracks with more severe AKI phenotypes and poorer short‐term outcomes; on postoperative day 3, cfDNA (threshold ~260 ng/mL) outperformed NGAL and creatinine for predicting late AKI (AUC = 0.804), whereas NGAL—though not cfDNA—was more closely associated with AKI stage and mortality in that cohort [23]. In trauma, cfDNA measured at admission discriminates non‐survivors from survivors and correlates with injury severity; ROC AUCs around 0.8 have been reported for early mortality prediction [62]. Together, these data indicate that cfDNA/mtDNA add prognostic information beyond conventional indices (creatinine, urine output, severity scores), capturing global injury burden and immune activation that are central to ICU‐AKI pathobiology.

## 4 Discussion

This mini‐review synthesizes current evidence on biomarkers spanning tubular injury/stress proteins and nucleic‐acid danger signals for ICU‐associated AKI. Several themes emerge. First, protein biomarkers—NGAL, KIM‐1, IL‐18, L‐FABP, and the cell‐cycle arrest pair [TIMP‐2]·[IGFBP7]—consistently detect renal stress or damage hours before creatinine/urine‐output criteria. Their strengths are organ proximity and biologic plausibility; their weaknesses are context effects (systemic inflammation, surgery, diuretics) and variability in thresholds across platforms and populations. Second, cfDNA/mtDNA capture a different dimension: The magnitude of cell death and innate immune activation at the organism level. Elevated levels track AKI development, dialysis need, and mortality in sepsis, cardiac surgery, and trauma, suggesting prognostic information that complements kidney‐specific markers rather than replaces them.

**FIGURE 2:**
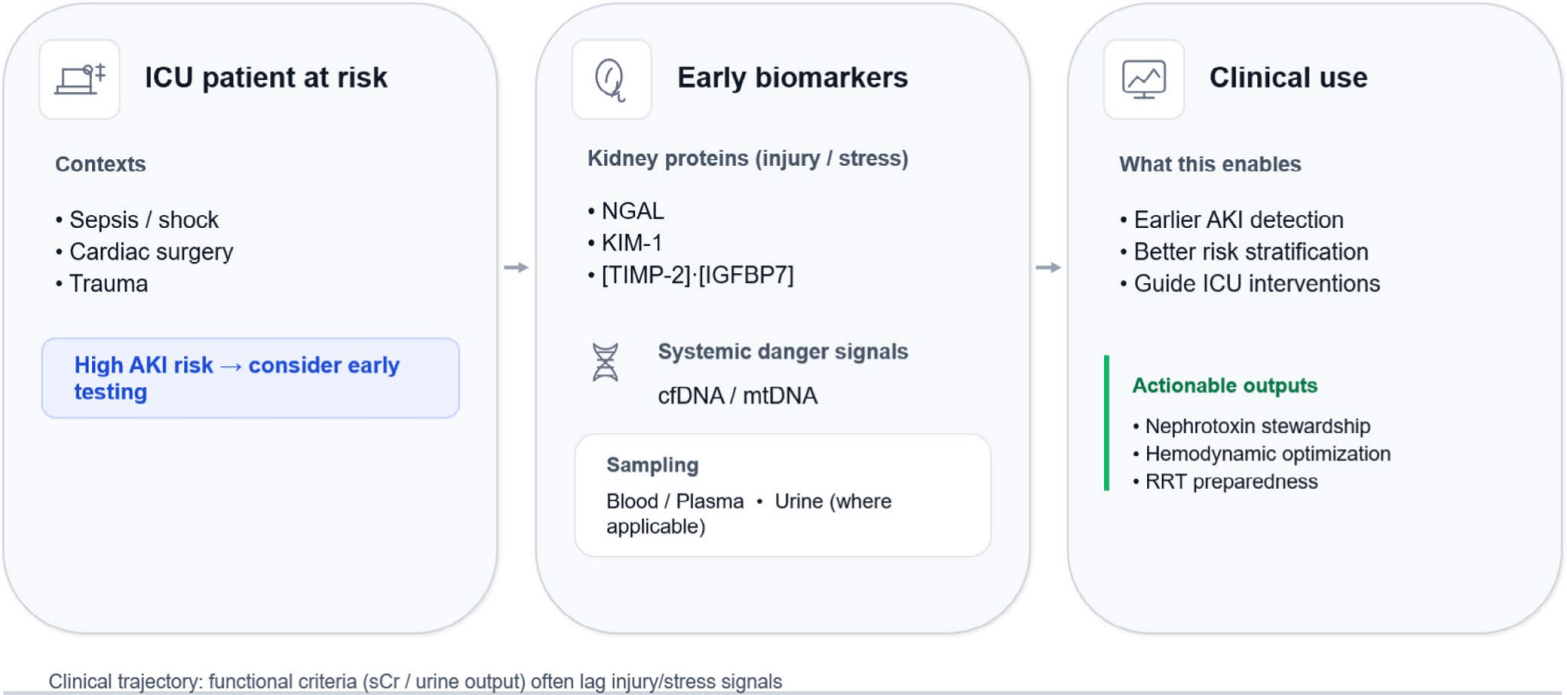
Proposed multimarker framework for ICU‐AKI.

Clinically, the differing kinetics and specificity profiles argue for a multimarker approach aligned to patient phenotype and decision points. NGAL rises very early after ischemic/toxic hits; [TIMP‐2]·[IGFBP7] marks a vulnerable “pre‐injury” window; cfDNA/mtDNA appear to better reflect evolving multi‐organ injury over subsequent days. Thus, algorithms that layer baseline risk and hemodynamics, an early kidney‐targeted marker, and cfDNA/mtDNA for systemic injury may widen the diagnostic window, enable earlier KDIGO bundle deployment, guide nephrotoxin stewardship, and prompt timely nephrology consultation or RRT planning (Figure 2).

However, translation requires confronting practical limitations. Pre‐analytical handling strongly influences nucleic‐acid measurements (anticoagulant choice, time‐to‐plasma, two‐step centrifugation, hemolysis control), and assay heterogeneity (antibody lots for proteins; qPCR targets/units for cfDNA/mtDNA) undermines cut‐off portability. Many studies are single‐center, with small samples and outcome‐ascertainment differences; few directly compare biomarker‐guided care vs. usual care. Costs and turnaround time also remain barriers outside research environments. In resource‐limited ICUs, limited access to rapid testing platforms, trained personnel, and laboratory infrastructure may constrain implementation and timely result reporting [38, 63]. In addition, reimbursement policies and insurance coverage vary across health systems and can be a key determinant of whether biomarker testing is adopted in routine care [64, 65].

In summary, moving “from NGAL to cfDNA” reflects a shift from single‐organ surrogates to systems‐level readouts. Carefully combined kidney‐specific proteins and cfDNA/mtDNA, embedded in actionable ICU pathways and measured under standardized conditions, offer the most credible route to earlier diagnosis and sharper prognostication in ICU‐AKI.

## Author Contributions

Conceptualization, Zhenhua Ji, Qi Zhu, and Hua Zheng; Methodology, Zhenhua Ji, Hua Zheng, and Xinxin Guo; Literature search and screening, Qi Zhu, Xinxin Guo, Limin Chen, Daoli Wang, Jiaqi Wang, Xinmeng Wang, and Zhiyuan Huang; Data extraction and evidence synthesis, Qi Zhu, Xinxin Guo, Zhijin Chen, and Limin Chen; Visualization (tables/figures), Feng Yang, Xinxin Guo, and Zhijin Chen; Validation/clinical interpretation, Zhenhua Ji, Hua Zheng, Daoli Wang, and Jiaqi Wang; Writing – original draft, Qi Zhu; Writing – review and editing, Zhenhua Ji, Hua Zheng, Feng Yang, Xinxin Guo, Zhijin Chen, Limin Chen, Daoli Wang, Jiaqi Wang, Xinmeng Wang, and Zhiyuan Huang; Supervision and project administration, Zhenhua Ji; Funding acquisition, not applicable. Zhenhua Ji is the guarantor and takes responsibility for the integrity of the work as a whole. All authors read and approved the final manuscript and agree to be accountable for all aspects of the work.

## Funding

This research is supported by the Liaoning Provincial Natural Science Foundation of China (2024‐BSLH‐291).

## Ethics Statement

The authors have nothing to report.

## Conflicts of Interest

The authors declare no conflicts of interest.

## Data Availability

Data sharing not applicable to this article as no datasets were generated or analysed during the current study.
